# A Rare Case of Rectal Carcinoma With Pulmonary Artery Thrombosis

**DOI:** 10.7759/cureus.56095

**Published:** 2024-03-13

**Authors:** Milly Mrinalini Tadigiri, Arub Imam, Rishab J Martins, Feral Daruwala

**Affiliations:** 1 Department of Surgery, Bharati Vidyapeeth Hospital, Pune, IND; 2 Freelance, Medical Writing, Surat, IND

**Keywords:** thromboembolism, deep vein thrombosis, pulmonary artery, thrombosis, rectal carcinoma

## Abstract

Cancer is a well-recognized risk factor for thromboembolic events and thromboembolism. This case report presents the rare coexistence of rectal carcinoma and pulmonary artery thrombosis in a female patient. A 44-year-old female presented with complaints of abdominal pain, vomiting, and obstipation. She had recently been diagnosed with rectal carcinoma. She had not undergone any invasive procedures in the past. Given the possibility of intestinal obstruction, an exploratory laparotomy was performed, followed by retrocolic gastrojejunostomy with Roux-en-Y anastomosis with a feeding JT tube insertion. On postoperative day six, she experienced symptoms of shortness of breath, tachypnoea, and hypoxia. At that time, a two-dimensional echocardiography showed normal findings but a CT pulmonary angiogram (CTPA) revealed a thrombus in the right upper lobe pulmonary artery. There was no evidence of lung metastasis on CTPA. This report highlights the challenging scenario associated with rectal carcinoma and concomitant pulmonary artery thrombosis.

## Introduction

Venous thromboembolism (VTE), encompassing deep vein thrombosis (DVT) and pulmonary thromboembolism (PTE), is a life-threatening condition. PTE ranks as the third leading cause of cardiovascular mortality, following acute coronary artery disease and stroke. PTE accounts for over three million deaths annually worldwide [1]. Following the first description of cancer-related thrombosis by Armand Trousseau, cancer-associated venous thrombosis has been increasingly recognized. Nowadays, cancer is considered a major risk factor for thromboembolic events and thromboembolism. We report a case of a 44-year-old female with a recent history of rectal carcinoma who was also diagnosed with pulmonary artery thrombosis. The coexistence of both these entities is reported in the literature, and its management can be complex and challenging.

## Case presentation

A 44-year-old Indian female presented with abdominal pain for seven days, vomiting for five days, and obstipation for four days. She had been recently diagnosed with rectal carcinoma [American Joint Committee on Cancer (AJCC) grade, IVC] and undergone four cycles of chemotherapy (400-600 mg/m^2^ per day), leucovorin (20 mg/m^2^ per day), and dexamethasone (10 mg/ml per day) along with 18 cycles of radiation therapy. Apart from this, she did not report any other comorbidities. She had left iliac tenderness with a soft abdomen. Cardiovascular, respiratory, and nervous system examinations were unremarkable. Hematological and biochemistry parameters were within normal limits. Two-dimensional (2D) echocardiography revealed an ejection fraction of 60%. Ultrasound showed right-sided hydronephrosis with proximal hydroureter due to distal ureteric obstruction. Contrast-enhanced CT (CECT) of the abdomen demonstrated circumferential asymmetric wall thickening at anorectum, with a maximum thickness of 1.1 cm, involving rectum length of 4.5 cm with mesorectal fat stranding, air focus within right lateral wall of thickened rectum, distorted fat planes in right levator ani muscle. There was mild right hydroureteronephrosis and abdominopelvic lymphadenopathy. She underwent exploratory laparotomy followed by retrocolic gastrojejunostomy with Roux-en-Y anastomosis with a feeding JT tube insertion. She remained stable during the immediate postoperative period.

**Figure 1:**
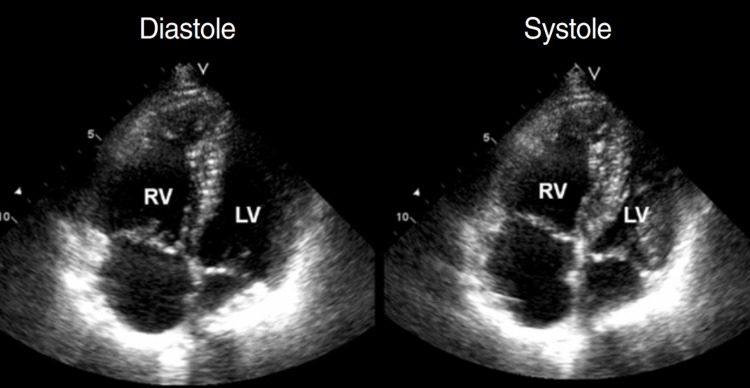
2D echocardiography of the patient The images show a dilated right atrium and right ventricle with suspicion of pulmonary embolism RV: right atrium; LV: left ventricle

**Figure 2:**
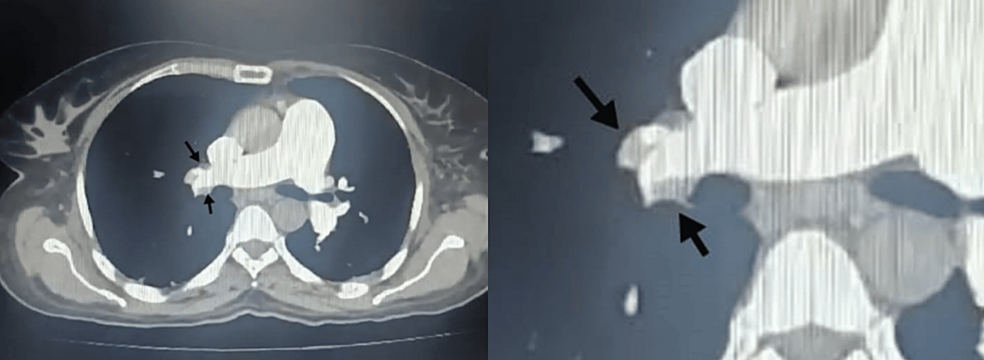
CTPA of the patient The black arrows show the right upper lobe pulmonary artery thrombus CTPA: computed tomography pulmonary angiogram

The patient had not been on any prophylactic anticoagulation. On postoperative day six, she developed shortness of breath, tachypnoea, and hypoxia. At that time, a 2D echocardiography was performed, which showed normal left ventricular systolic function with dilated right atrium, right ventricle, and moderate tricuspid valve regurgitation with severe pulmonary artery hypertension (Figure 1). CT pulmonary angiogram (CTPA) revealed a thrombus in the right upper lobe pulmonary artery (Figure 2). There was no evidence of lung metastasis on CTPA. She was started on unfractionated heparin, followed by oral anticoagulant rivaroxaban. Her condition gradually improved and she was discharged on oral anticoagulation and home oxygen therapy with minimal oxygen requirement varying from 2 to 4 liter/minute.

## Discussion

The annual incidence of VTE in cancer patients is currently estimated to be 0.5%, compared to 0.1% in the general population. Active cancer contributes to 20% of the overall incidence of VTE [2-3]. Among different cancers, hematological malignancies are the most common cause of VTE, followed by lung and gastrointestinal cancers [2]. Colorectal cancers are the third most commonly diagnosed cancer, with an increasing incidence of associated morbidity and mortality [4].

Cancer patients suffer from several conditions that predispose them to thrombus generation. The process of thrombus formation in cancer patients differs from that in individuals without cancer. Several factors play a role in the immune response to neoplasia, such as the development of acute phase reactants, abnormal protein metabolism, necrosis, and hemodynamic rearrangements. These factors collectively contribute to the overall activation of blood coagulation in cancer patients. Nevertheless, a significant role is attributed to tumor-specific prothrombotic mechanisms, encompassing various properties of tumor cells [2,5]. Malignant cells can interact with the hemostatic system through various mechanisms, with two main types of interaction being (1) the ability to generate and release procoagulant and fibrinolytic substances, or 2) tumor and host cell interaction [2,5].

Older age [6] and female sex are considered risk factors for thrombus formation. Aside from these, ethnic factors, major comorbidities like heart disease, renal failure, obesity, acute infection, prolonged immobility, and prior history of thromboembolism increase the risk of pulmonary embolism [2,3,5]. Certain cancer-related risk factors also impact the development of thrombus formation. As per recent data, pancreatic cancer carries the maximum risk of thromboembolism among gastrointestinal cancers. Apart from the site of cancer, an advanced stage of malignancy and cancer with distant metastases substantially increases the risk of VTE than the early stage of disease and local or regional metastasis. Cohort studies have shown that the risk of developing VTE heightened with the advancement of the cancer stage. The corresponding calculated adjusted relative risks for stage I, II, III, and IV were reported to be 2.9, 2.9, 7.5, and 17.1, respectively [3,7]. Similarly, patients with high-grade tumors (G3 or G4) are more likely to develop VTE. Among all histological variants, mucin-producing adenocarcinoma is linked with the highest risk of VTE among gastrointestinal cancers [3,8].

The immediate period following diagnosis carries a significantly high risk of VTE because many therapeutic interventions are performed during this period, including chemoradiation and surgical treatment, which itself increases the risk of VTE. The highest incidence of VTE occurs within the first six months following a cancer diagnosis, with a subsequent decline in the risk of VTE [3]. Cancer-related surgery of the pelvis and abdomen is linked to an increased risk of VTE development. Chemotherapy causes a six-to-sevenfold increase in the chance of VTE in cancer patients. Due to the use of many chemotherapeutic agents and the inclusion of steroids, chemotherapy increases the risk of VTE. The placement of a central venous catheter in the postoperative ICU, particularly in the femoral vein, also increases the risk of VTE. Anorectal carcinoma is a common variant of solid organ malignancies, and it carries a high risk of grave complications like VTE and pulmonary hypertension.

Our patient had several risk factors, such as female sex, the presence of advanced malignancy with high-grade and distant metastasis, adenocarcinoma variant, a recent history of chemoradiation, a previous history of abdominal pelvic surgery, and prolonged immobilization, which explains her increased risk of VTE with complications of pulmonary embolism.

## Conclusions

We discussed a rare occurrence of rectal carcinoma coexisting with pulmonary artery thrombosis. Factors like advanced malignancy, distant metastasis, high-grade tumors, recent chemoradiation, and surgical history are all believed to contribute to an increased risk of VTE. This report underscores the importance of promptly recognizing and managing thrombotic complications in patients with rectal carcinoma to ensure timely intervention and improved patient outcomes.
